# The impact of expanded telehealth availability on primary care utilization

**DOI:** 10.1038/s41746-022-00685-8

**Published:** 2022-09-09

**Authors:** Ram A. Dixit, Raj M. Ratwani, Jasmine A. Bishop, Kevin Schulman, Christopher Sharp, Kerry Palakanis, Ethan Booker

**Affiliations:** 1 MedStar Health National Center for Human Factors in Healthcare, Washington, DC USA; 2 MedStar Health Telehealth Innovation Center, Washington, DC USA; 3 Stanford Medicine, Palo Alto, CA USA; 4 Intermountain Healthcare, Salt Lake City, UT USA

**Keywords:** Health services, Health policy

## Abstract

The expanded availability of telehealth due to the COVID-19 pandemic presents a concern that telehealth may result in an unnecessary increase in utilization. We analyzed 4,114,651 primary care encounters (939,134 unique patients) from three healthcare systems between 2019 and 2021 and found little change in utilization as telehealth became widely available. Results suggest telehealth availability is not resulting in additional primary care visits and federal policies should support telehealth use.

During the COVID-19 public health emergency in the United States, federal waivers have allowed all beneficiaries to receive care via telehealth anywhere it was needed, removing rural and facility originating site restrictions and allowing billing to be guided by level of service comparable to in-person care^1,2^. These changes permitted rapid expansion of telehealth, which addressed patients’ need for care while minimizing pandemic-associated risks^3,4^. This response now provides an opportunity to address a longstanding concern that provision of telehealth services would result in an unnecessary increase in utilization without impacting patient outcomes. Understanding whether expanded telehealth use increases utilization has important implications for policy and reimbursement.

We sought to determine whether there was an increase in primary care utilization with the expanded availability of telehealth by analyzing 4,114,651 primary care encounters from 939,134 unique patients across three healthcare systems.

The mean number of encounters from 2019 to 2021 for all patients was 2.30 (SD 1.91), 2.26 (SD 1.92), and 2.27 (SD 1.89). Looking at the subset of patients that had at least one encounter every year, termed “matched” patients, the mean number of encounters from 2019 to 2021 was 2.69 (SD 2.11), 2.69 (SD 2.20), and 2.63 (SD 2.15). The mean number of encounters for all patients and matched patients by payor type (commercial, Medicaid, Medicare, and other; Fig. 1) showed a similar pattern of relatively little change from year to year. For all patients and matched patients, those patients that had more than one encounter per year tended to use telehealth more than those with only one encounter per year (Fig. 2).

**Fig. 1:**
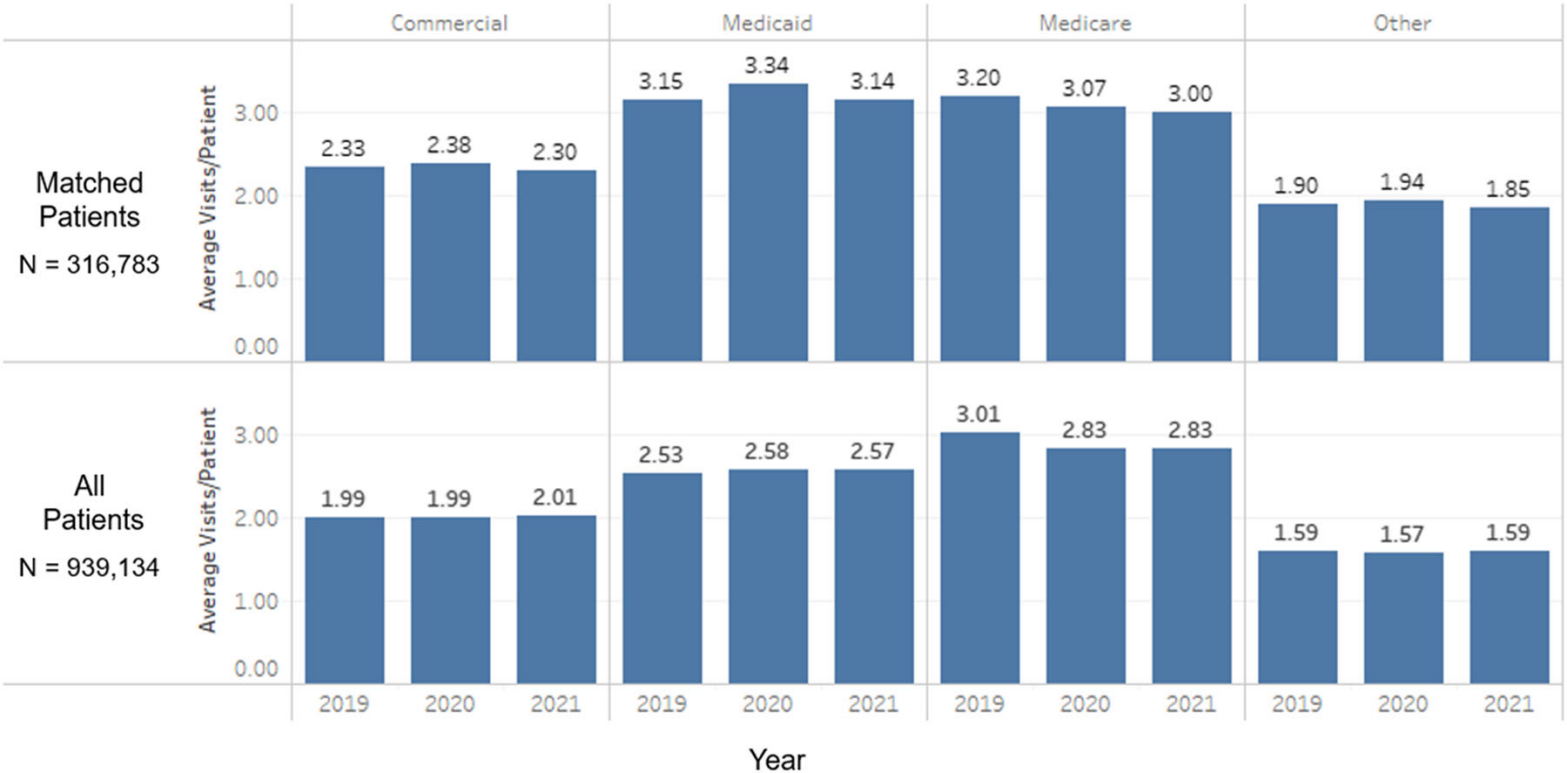
Average number of primary care visits per patient remain stable from 2019 to 2021 across insurance groups. This figure shows the average number of encounters per year for all patients and matched patients by payor type. The number of patients in each insurance category are as follows: Commercial (621,490 total; 176,543 matched), Medicaid (74,853 total; 20,050 matched), Medicare (225,575 total; 128,137 matched), Other (42,306 total; 7291 matched).

**Fig. 2:**
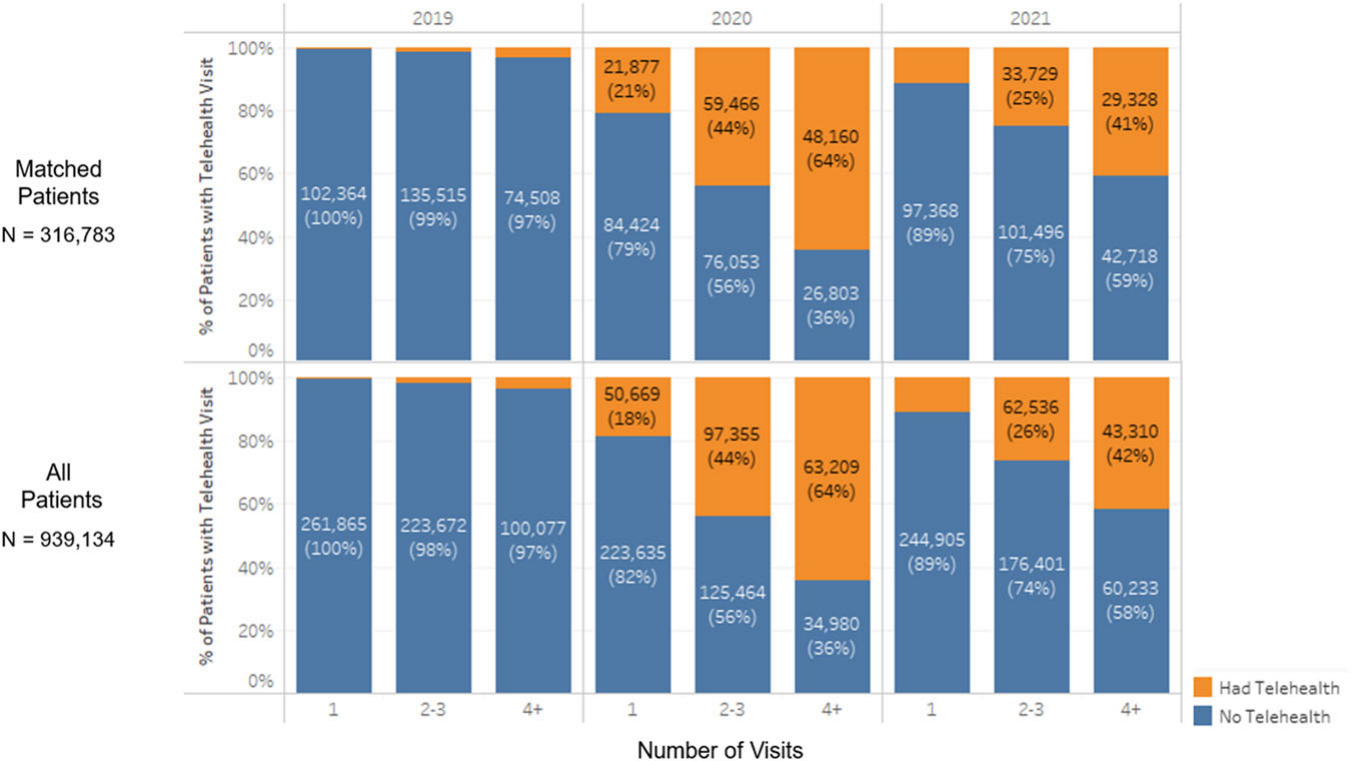
Telehealth use occurs more in patients with multiple primary care visits. Number and percent of patients with no (blue) or at least one telehealth visit (orange) grouped by number of primary care appointments in that year for matched patients (top) and all patients (bottom).

Our results suggest the availability of telehealth is not resulting in additional primary care visits, rather, telehealth is serving as a substitute for certain in-person encounters resulting in no overall increase in primary care utilization. Further, it seems telehealth was mostly utilized for patients whose medical needs required multiple primary care visits during each year, suggesting that these telehealth encounters enabled follow-up for patients with chronic illness^5,6^. Future work can address whether expanded access to primary care services had downstream benefits in terms of utilization of other services such as urgent care clinics or emergency departments^7^. However, when looking at primary care, these data seem to refute the concern about inducing unnecessary utilization because of expanded access to care.

This study has several limitations. Patients may have received additional primary care services from other healthcare facilities that are not included in the dataset. Also, we could not assess the quality of the encounter to compare in-person to telehealth. Patients using either care modality may have received additional care in an emergency department or urgent care clinic, and this was not accounted for in our analysis. Overall primary care utilization may have been suppressed due to the pandemic blunting the effect of telehealth. Overall, however, these data are reassuring that expansion of telehealth services maintained access during the pandemic without increasing overall quantity of services for a large primary care population.

## Methods

Completed in-person and telehealth (video or phone) primary care encounters for adults (age ≥ 18 years) at MedStar Health, Stanford Health Care, and Intermountain Healthcare from January 1, 2019 to December 31, 2021 were analyzed with each patient’s payor type (Commercial, Medicaid, Medicare, and Other). Primary care was defined as scheduled outpatient encounters with primary care physicians consisting of internal medicine, family medicine, and geriatrics. Patients whose payor type changed within a year were excluded from all analyses and consisted of less than 4% of patients (*N* = 35,906). Data were analyzed in two ways to determine the mean number of encounters per patient per year and to identify telehealth use. One analysis was performed on all encounters, consisting of 4,114,651 encounters from 939,134 patients without consideration for whether the same patient was seen year over year. A second analysis was performed with a subset of the data consisting of only patients that had at least one encounter in each of the three years, termed “matched” patients consisting of 2,540,158 encounters from 316,783 patients. The number of patients in each insurance category are as follows: Commercial (621,490 total; 176,543 matched), Medicaid (74,853 total; 20,050 matched), Medicare (225,575 total; 128,137 matched), Other (42,306 total; 7291 matched). This study was approved by the MedStar Health Research Institute, Stanford Health Care, and Intermountain Health institutional review boards and the need for informed consent was waived.

### Reporting summary

Further information on research design is available in the Nature Research Reporting Summary linked to this article.

## Supplementary information

Reporting Summary Checklist

## Data Availability

The data analyzed in this study were combined across three healthcare systems used through mutual Data Use Agreements and under Institutional Review Board approval, hence, they are not publicly available. Further details or inquiry into the dataset can be provided upon reasonable request and with permission from each healthcare institution. Interested parties can contact the corresponding author Raj Ratwani at Raj.M.Ratwani@MedStar.net.
