# Supplementary material for: The impact of expanded telehealth availability on primary care utilization
Source: NPJ Digit Med. 2022 Sep 9;5:141. doi: 10.1038/s41746-022-00685-8 (PMC9462602; doi:10.1038/s41746-022-00685-8)
Supplement: Supplementary file 1 — Reporting Summary Checklist [file 41746_2022_685_MOESM1_ESM.pdf]

nature portfolio

Corresponding author(s): Raj Ratwani

Last updated by author(s): Aug 1, 2022

Reporting Summary

Nature Portfolio wishes to improve the reproducibility of the work that we publish. This form provides structure for consistency and transparency in reporting. For further information on Nature Portfolio policies, see our Editorial Policies and the Editorial Policy Checklist.

Statistics

For all statistical analyses, confirm that the following items are present in the figure legend, table legend, main text, or Methods section.

n/a	Confirmed	
☐	☒	The exact sample size (*n*) for each experimental group/condition, given as a discrete number and unit of measurement
☐	☒	A statement on whether measurements were taken from distinct samples or whether the same sample was measured repeatedly
☒	☐	The statistical test(s) used AND whether they are one- or two-sided<br>*Only common tests should be described solely by name; describe more complex techniques in the Methods section.*
☒	☐	A description of all covariates tested
☒	☐	A description of any assumptions or corrections, such as tests of normality and adjustment for multiple comparisons
☒	☐	A full description of the statistical parameters including central tendency (e.g. means) or other basic estimates (e.g. regression coefficient) AND variation (e.g. standard deviation) or associated estimates of uncertainty (e.g. confidence intervals)
☒	☐	For null hypothesis testing, the test statistic (e.g. *F*, *t*, *r*) with confidence intervals, effect sizes, degrees of freedom and *P* value noted<br>*Give P values as exact values whenever suitable.*
☒	☐	For Bayesian analysis, information on the choice of priors and Markov chain Monte Carlo settings
☒	☐	For hierarchical and complex designs, identification of the appropriate level for tests and full reporting of outcomes
☒	☐	Estimates of effect sizes (e.g. Cohen's *d*, Pearson's *r*), indicating how they were calculated

Our web collection on statistics for biologists contains articles on many of the points above.

Software and code

Policy information about availability of computer code

Data collection	Data were extracted from each site's electronic health record system.
Data analysis	Tableau 2021.2 was used for basic descriptive analysis of the data.

For manuscripts utilizing custom algorithms or software that are central to the research but not yet described in published literature, software must be made available to editors and reviewers. We strongly encourage code deposition in a community repository (e.g. GitHub). See the Nature Portfolio guidelines for submitting code & software for further information.

Data

Policy information about availability of data

All manuscripts must include a data availability statement. This statement should provide the following information, where applicable:

- Accession codes, unique identifiers, or web links for publicly available datasets
- A description of any restrictions on data availability
- For clinical datasets or third party data, please ensure that the statement adheres to our policy

The data analyzed in this study were combined across three healthcare systems used through mutual Data Use Agreements and under Institutional Review Board approval; hence, they are not publicly available. Further details or inquiry into the dataset can be provided upon reasonable request and with permission from each healthcare institution.

Human research participants

Policy information about studies involving human research participants and Sex and Gender in Research.

Reporting on sex and gender	This data was not collected and analyzed for this study.
Population characteristics	Insurance information for patients was extracted at each site and categorized into four categories (Commerical Insurance, Medicare, Medicaid, and Other).
Recruitment	n/a
Ethics oversight	MedStar Health Research Institute, Stanford Health Care, Intermountain Health

Note that full information on the approval of the study protocol must also be provided in the manuscript.

Field-specific reporting

Please select the one below that is the best fit for your research. If you are not sure, read the appropriate sections before making your selection.

☐ Life sciences ☒ Behavioural & social sciences ☐ Ecological, evolutionary & environmental sciences

For a reference copy of the document with all sections, see nature.com/documents/nr-reporting-summary-flat.pdf

Behavioural & social sciences study design

All studies must disclose on these points even when the disclosure is negative.

Study description	Quantitative Longitudinal Analysis
Research sample	Adult patients at MedStar Health, Stanford Health Care, and Intermountain Health who utilized primary care from 2019-2021.
Sampling strategy	Data were not sampled - all primary care appointments were included for analysis.
Data collection	Data were extracted from each site's electronic health record system.
Timing	Jan 1, 2019 - Dec 31, 2021
Data exclusions	Patients whose insurance status changed within a calendar year were excluded from the study.
Non-participation	N/A
Randomization	N/A

Reporting for specific materials, systems and methods

We require information from authors about some types of materials, experimental systems and methods used in many studies. Here, indicate whether each material, system or method listed is relevant to your study. If you are not sure if a list item applies to your research, read the appropriate section before selecting a response.

Materials & experimental systems

n/a	Involved in the study	
☒	☐	Antibodies
☒	☐	Eukaryotic cell lines
☒	☐	Palaeontology and archaeology
☒	☐	Animals and other organisms
☒	☐	Clinical data
☒	☐	Dual use research of concern

Methods

n/a	Involved in the study	
☒	☐	ChIP-seq
☒	☐	Flow cytometry
☒	☐	MRI-based neuroimaging
